# A Comprehensive Architecture for Federated Learning-Based Smart Advertising

**DOI:** 10.3390/s24123765

**Published:** 2024-06-09

**Authors:** Rasool Seyghaly, Jordi Garcia, Xavi Masip-Bruin

**Affiliations:** UPC BarcelonaTECH, CRAAX Laboratory, 08800 Vilanova, Spain; jordi.garcia@upc.edu

**Keywords:** federated learning, smart advertising, data architecture, privacy preservation, edge computing, data validation, machine learning models, user engagement, personalized advertisements, network resource consumption

## Abstract

This paper introduces a cutting-edge data architecture designed for a smart advertising context, prioritizing efficient data flow and performance, robust security, while guaranteeing data privacy and integrity. At the core of this study lies the application of federated learning (FL) as the primary methodology, which emphasizes the authenticity and privacy of data while promptly discarding irrelevant or fraudulent information. Our innovative data model employs a semi-random role assignment strategy based on a variety of criteria to efficiently collect and amalgamate data. The architecture is composed of model nodes, data nodes, and validator nodes, where the role of each node is determined by factors such as computational capability, interconnection quality, and historical performance records. A key feature of our proposed system is the selective engagement of a subset of nodes for modeling and validation, optimizing resource use and minimizing data loss. The AROUND social network platform serves as a real-world case study, illustrating the efficacy of our data architecture in a practical setting. Both simulated and real implementations of our architecture showcase its potential to dramatically curtail network traffic and average CPU usage, while preserving the accuracy of the FL model. Remarkably, the system is capable of achieving over a 50% reduction in both network traffic and average CPU usage even when the user count escalates by twenty-fold. The click rate, user engagement, and other parameters have also been evaluated, proving that the proposed architecture’s advantages do not affect the smart advertising accuracy. These findings highlight the proposed architecture’s capacity to scale efficiently and maintain high performance in smart advertising environments, making it a valuable contribution to the evolving landscape of digital marketing and FL.

## 1. Introduction

This research lies in the intersection of two key domains: smart advertising and FL. Smart advertising refers to the use of advanced technologies and data-driven strategies to deliver personalized and targeted advertisements to users. With the proliferation of digital platforms and the availability of vast amounts of user data, smart advertising has gained significant traction as an effective marketing approach. However, challenges such as data privacy, resource consumption, and maintaining data integrity hinder the optimal implementation of smart advertising.

Smart advertising represents a transformative shift in the marketing landscape. By utilizing the capabilities of data and AI, advertisers can deliver more personalized and relevant content to users, leading to improved engagement and better campaign performance. However, responsible data usage and privacy considerations are crucial to ensuring the sustainability and success of smart advertising in the long run [1].

On the other hand, FL now stands as a promising approach for decentralized machine learning, where models are trained collaboratively on local devices without the need for centralized data aggregation. FL tackles issues concerning data privacy and security by allowing organizations to harness the combined knowledge from decentralized data sources while upholding user privacy. However, the implementation of FL in smart advertising requires a tailored data architecture that addresses the specific challenges and requirements of this domain.

Edge computing is an emerging paradigm that brings data processing and storage closer to the edge of the network, enabling real-time processing and reducing latency by distributing computing resources to edge nodes. It addresses the limitations of the centralized cloud infrastructure and offers benefits such as improved responsiveness, reduced network congestion, and enhanced security and privacy. Edge computing finds applications in areas requiring real-time decision-making, and its growth is driven by its ability to enable innovative services and applications in the Internet of Things (IoT) and the Fourth Industrial Revolution. However, it also poses challenges such as resource constraints, data management, scalability, and security.

Edge computing plays a crucial role in facilitating FL by offering the essential infrastructure required for the decentralized processing of data across edge devices. FL has the potential to address the limitations of the centralized cloud infrastructure, such as latency and bandwidth constraints, by capitalizing on the close proximity of edge nodes to data sources. The integration of edge computing and FL not only improves operational effectiveness but also guarantees the confidentiality and protection of data at the network’s edge. In this study we will incorporate edge technology to leverage FL.

In the rapidly evolving landscape of digital marketing, the convergence of smart advertising and FL holds immense significance. A comprehensive architecture that marries these two domains can unlock a multitude of benefits and address critical challenges.

Firstly, the synergy between smart advertising and FL has the potential to revolutionize the way advertisers approach their target audience. The personalized nature of smart advertising can be further refined using the collaborative learning approach of FL. This can lead to hyper-personalized recommendations and advertisements that cater to individual preferences and behaviors, ultimately enhancing user experience and engagement.

Secondly, the issue of data privacy, which is central to both domains, can be effectively tackled through a well-designed architecture. The decentralized model of FL ensures that user data remain on their devices, never needing to be centralized. This not only alleviates privacy concerns but also fosters user trust, a vital component of successful advertising campaigns.

Thirdly, resource consumption and efficiency can be optimized by leveraging the distributed nature of FL. Advertisers can tap into the computational power of various devices while minimizing the strain on any single device or server. This dynamic allocation of resources can lead to faster model training and advertisement refinement, translating to quicker campaign deployment and adaptability to changing market trends.

Furthermore, a comprehensive architecture for smart advertising within the FL framework can lead to a more accountable and transparent advertising ecosystem. With mechanisms to verify the accuracy of advertisements and ensure data integrity, stakeholders can have confidence in the effectiveness of their campaigns, fostering long-term sustainability.

This research lies in the need to bridge the gap between smart advertising and FL by developing a comprehensive architecture. By understanding the unique challenges of smart advertising, such as security concerns, accuracy of advertisements, and resource consumption, this research aims to propose a comprehensive architecture for smart advertising based on FL.

Motivations of this research stem from the growing significance of smart advertising and the need for an optimized data architecture and accurate modeling in the context of FL. Smart advertising, driven by advancements in technology and data-driven insights, offers personalized and targeted ad experiences to users. However, accurate modeling, achieving efficient data flow, maintaining data integrity, ensuring privacy, and minimizing resource consumption are significant challenges in this area.

This research focuses on a comprehensive architecture for utilizing federated learning (FL) in the context of smart advertising, including real-world testing with actual users. The real-world assessment has been conducted on the AROUND social network, which boasts a user base exceeding 3 million. The main contributions of this research can be summarized as follows:

- A sophisticated recommender system using FL for the accurate modeling of advertisements which is employed to select the most appropriate business to be suggested to customers.
- An edge-based data model and architecture to keep response time fast, control resource consumption, and handle data integrity.
- A robust interference recognition system to ensure the privacy and security.

While numerous studies have delved into the realms of sophisticated recommendation systems, edge-based data models, and privacy-centric interference recognition, this architecture stands as a testament to the power of synthesis. By simultaneously embracing and interweaving these multifarious objectives, this research transcends the limitations of isolated advancements. It recognizes that in the intricate dance between accurate recommendation modeling, efficient resource utilization, rapid responsiveness, and unwavering data security, the true potential of smart advertising within the framework of FL emerges.

The research presents a novel architecture for smart advertising based on edge-based FL, showcasing its impact on system performance, scalability, and user engagement. We introduce a sophisticated recommender system, an edge-based data model, and a robust interference recognition system.

The proposed system exhibits the efficient handling of network traffic, decreased CPU usage with increased nodes, improved prediction accuracy with federated aggregation, and enhanced user engagement with reduced opt-out rates. These achievements are vividly illustrated in the results presented in this paper.

Federated learning-based smart advertising research shows how scientific fields can work together to solve complex problems. This study creates a cutting-edge data architecture for smart advertising by integrating computer science, data architecture, machine learning, and digital marketing. Computer science lays the groundwork for data processing and algorithm development, while federated learning offers innovative privacy-preserving data analysis methods. Smart advertising applications need data architecture expertise for efficient data flow and performance optimization. Digital marketing insights also improve advertising relevance and effectiveness by providing domain-specific knowledge. Using the strengths of each field, this interdisciplinary collaboration creates a comprehensive architecture that meets smart advertising’s unique needs while ensuring data integrity, privacy, and high performance.

In the rest of this paper, a comprehensive review of related works available in the literature is presented in Section 2. Following that, Section 3 elucidates the contextual terms and concepts pertinent to the study. The AROUND project, its objectives, and significance are then introduced in Section 4. The proposed method, which stands as a key contribution of this research, is expounded upon in Section 5 alongside a description of the core reccomender system in Section 6. The obtained results and their analysis are subsequently presented in Section 7. Finally, the research is concluded and summarized in Section 8, providing a comprehensive overview of the findings and their implications.

## 2. Related Works

This section presents a selection of works related to this research, focusing on various aspects of the comprehensive architecture. We begin by exploring studies concerning smart advertising and edge-based strategies, followed by an examination of research pertaining to FL and data models for smart advertising tailored for this domain.

Kim and Lee [2] proposed that the mobile advertising paradigm is evolving towards a personalized and context-aware approach to advertisement services for individual consumers. Their study aimed to identify different customer typologies by combining the Q method and R empirical methods. Subsequently, these typologies were employed as fundamental statistical data for the domains of advertising marketing and customer relationship management.

Also, Verhoef et al. [3] observed that modern consumers are deeply embedded in extensive and intricate networks, comprising interconnected individuals. They introduced the POP framework, which explores the interconnectedness between People, Objects, and the Physical world. Their study aimed to investigate the plausible influence of the IoT and smart products on consumer behavior and firm strategies.

The study by Jin et al. [4] focuses on optimizing real-time bidding in advertising through reinforcement learning techniques. It addresses the dynamic nature of ad auctions and aims to achieve optimal bidding strategies that adapt to changing market conditions.

Naumov et al. [5] introduced personalized ad recommendation systems that leverage user behavior data and deep learning. It emphasizes the importance of providing relevant ads to users to improve click-through rates and overall advertising effectiveness.

Helberger et al. [6] recognized the significant transformations occurring in the creation, targeting, and delivery of advertising messages. They observed that smart advertising is reshaping the roles of consumers, leading them to actively engage with brands and become creators of ad content. Additionally, consumers now play an active role in distributing advertisements through interpersonal networks, effectively becoming influential advertisers themselves. This shift highlights the increased influence and power of consumers in the advertising landscape compared to previous times.

These studies exemplify the ongoing efforts to enhance the efficiency and personalization of mobile advertising systems through cutting-edge machine learning and AI techniques. By addressing critical challenges and leveraging diverse algorithms, researchers aim to pave the way for more effective and contextually relevant mobile advertising experiences.

There are also some related works in terms of FL and data model research. Smith and Johnson [7] proposed an optimized data architecture for FL in IoT networks. The study focuses on data aggregation and compression techniques to reduce communication overhead and improve learning efficiency.

Johnson and Anderson [8] presented a data sharing approach using homomorphic encryption in FL. The work addressed data privacy concerns while allowing the secure aggregation of encrypted model updates.

The research by Chen et al. [9] explored data filtering and feature extraction techniques for FL in image recognition tasks. The study aimed to reduce data dimensionality and optimize model training with limited communication resources.

Also, Li et al. [10] proposed an optimization framework for data partitioning and scheduling in FL for edge computing environments. The work considered resource constraints and network latency to improve learning efficiency.

Wang et al. [11] addressed privacy concerns in healthcare data sharing for FL. The study introduced a privacy-preserving data aggregation mechanism that protects sensitive patient information while enabling collaborative model training.

Zhang et al. [12] focused on data integration techniques for personalized recommendation in FL. The work explored data selection, aggregation, and model adaptation strategies to enhance recommendation performance.

Liu et al. [13] investigated data communication optimization techniques in FL with bandwidth awareness. The study considers network conditions and bandwidth constraints to improve communication efficiency.

In a recent study by Viktoratos and Tsadiras [14], the researchers tackled the cold-start problem in personalized mobile advertisement systems. The primary objective of their study was to tackle the challenge of making accurate predictions in scenarios where users possess minimal or no data accessible within the recommender system. To overcome this issue, they proposed a novel prediction approach that leverages various machine learning techniques, including logistic regression, support vector machines, as well as deep learning methods like FLEN and DeepFM.

Likewise, in their study, Ferro-Díez et al. [15] delved into the potential of artificial intelligence-enabled location-based services for mobile advertising. They scrutinized various algorithms, including random forest, support vector machines, and artificial neural networks, aiming to construct an efficient visualization of geographic areas alongside their relevance scores for a predetermined set of categories.

The work conducted by Kim et al. [16] introduced a novel architectural concept termed as blockchained federated learning (BlockFL). In this approach, exchanges and verifications of local learning model updates occur within a blockchain framework. This innovation facilitates on-device machine learning, eliminating the need for centralized training data or coordination. This is achieved through the implementation of a consensus mechanism within the blockchain structure. Furthermore, the study delves into the investigation of an end-to-end latency model for BlockFL. Notably, the research includes an analysis that characterizes the optimal rate of block generation. This characterization takes into account factors such as communication, computation, and consensus delays.

Stripelis et al. [17] presented a data-sharing approach using homomorphic encryption in FL to address data privacy concerns. Also, Grama et al. [18] introduced a privacy-preserving data aggregation mechanism in healthcare FL.

In Wang et al. [19], the authors utilized data as a flexible parameter to orchestrate training schedules, thereby achieving near-optimal solutions for computation time and accuracy loss. Drawing insights from offline profiling, optimization problems are formulated, and polynomial-time algorithms are proposed. This approach is tailored for both class-balanced and class-unbalanced data scenarios. The efficacy of the optimization framework is rigorously assessed through extensive evaluations conducted on a mobile testbed featuring two datasets.

The pioneering work by Xie et al. [20] introduced an innovative approach involving a novel multi-center aggregation mechanism designed for clustering clients based on their models’ parameters. This mechanism entails learning multiple global models, effectively utilizing them as the cluster centers. Concurrently, it identifies the optimal matching between users and these centers. The methodology further formulates this process into an optimization challenge, effectively addressed through the application of a stochastic expectation maximization (EM) algorithm. In a series of experiments carried out on various benchmark datasets of FL, the presented method consistently demonstrates its superiority over several prominent baseline techniques.

While these works provide valuable insights, they also exhibit certain limitations. Therefore, there remains a need for methods to effectively control and manage the overhead of processes on user devices, all while ensuring accurate engagement with advertisements. In this study, we aim to address these challenges with our proposed solutions.

## 3. Research Context

### 3.1. Smart Advertising

Smart advertising is a modern and data-driven approach to marketing that leverages advanced technologies to deliver personalized and engaging content to users. Unlike traditional advertising, which relies on broad targeting and mass distribution, smart advertising utilizes sophisticated algorithms and machine learning techniques to analyze vast amounts of user data. This enables advertisers to tailor their promotional messages based on individual preferences, behavior, and demographics [21].

The key to the success of smart advertising lies in its ability to provide relevant and timely content to users, enhancing the overall ad experience. Through comprehension of user interests and behavior, intelligent advertising can deliver tailored ads that resonate with the audience, thereby driving increased engagement and conversion rates.

Central to smart advertising is the use of data analytics and artificial intelligence (AI) algorithms. These technologies enable advertisers to extract valuable insights from user data, such as browsing history, purchase behavior, and online interactions. Through predictive modeling, advertisers can anticipate user preferences and deliver personalized ads that are more likely to generate interest and action.

Moreover, smart advertising extends beyond just online platforms. It incorporates various channels such as social media, mobile apps, and connected devices to reach users wherever they are present. This omnichannel approach ensures that advertisers can connect with their target audience at multiple touchpoints, creating a cohesive and seamless brand experience.

Despite its benefits, smart advertising also raises concerns about data privacy and consumer consent. Advertisers must strike a delicate balance between personalization and respecting user privacy rights. Implementing strong data protection measures and obtaining explicit user consent are essential to building trust and maintaining ethical advertising practices.

As smart advertising relies on collecting and analyzing user data to tailor ad experiences, ensuring data privacy becomes paramount. Advertisers should prioritize the implementation of robust security measures to safeguard user information from unauthorized access, breaches, or misuse. Techniques like encryption and secure data storage can help protect sensitive user data throughout its lifecycle.

Moreover, data minimization practices should be followed, wherein only necessary data for ad personalization are collected, reducing the risk associated with storing excessive user information. Periodic audits and assessments of data handling processes can help identify vulnerabilities and ensure compliance with privacy regulations.

Obtaining explicit and informed user consent is a cornerstone of ethical smart advertising. Advertisers should clearly communicate to users what data are being collected, how they will be used, and for what purposes. Consent should be voluntary, unambiguous, and granular, allowing users to choose the specific types of data processing they agree to.

Transparency plays a crucial role in building trust. Advertisers should provide accessible and easily understandable privacy policies that outline their data practices. This empowers users to make informed decisions about sharing their data and participating in personalized advertising.

Respecting user autonomy includes offering mechanisms for users to control their data. Advertisers should provide easy-to-use opt-out options, enabling users to withdraw their consent for data collection and personalized advertising at any time. Users should have the right to access, rectify, or delete their data if they choose to do so.

### 3.2. Federated Learning for Smart Advertising

Federated learning (FL) is a decentralized machine learning methodology that enables model training on local data without centralization. This innovative approach addresses critical privacy concerns by enabling training on local data stored on devices such as smartphones, IoT devices, and edge servers. By leveraging this distributed paradigm, FL ensures that sensitive data remain on the devices, safeguarding user privacy and data security. This decentralized nature not only enhances privacy but also minimizes the risks associated with data breaches and unauthorized access, making FL a promising solution for industries dealing with sensitive information.

The fundamental principle of FL lies in its iterative process, characterized by successive rounds of local model training and global model aggregation. In each round, individual devices autonomously update their local models using their data before sending only the model updates to a central server for aggregation. Through this collaborative learning mechanism, the global model evolves by incorporating the knowledge from diverse local datasets without direct data exchange. This federated approach not only preserves data privacy but also allows for continuous model improvement across distributed devices, enabling the creation of robust and generalized models.

Moreover, the versatility of FL extends beyond privacy preservation to scalability and efficiency enhancements. By distributing the training process among multiple devices, FL mitigates the computational burden on centralized servers, leading to faster model convergence and reduced communication costs. This decentralized framework paves the way for collaborative learning scenarios in resource-constrained environments, where data privacy and limited network bandwidth are paramount concerns. In essence, FL emerges as a promising avenue for advancing machine learning paradigms towards a more privacy-preserving, efficient, and scalable future.

In line with its versatile nature and potential benefits across various domains, including smart advertising, FL presents several promising applications in the field of smart advertising, enabling efficient and privacy-preserving ad targeting and personalization. These subsection explores some key applications of FL in the context of smart advertising.

#### 3.2.1. Privacy-Preserving Ad Personalization

FL allows for the training of personalized ad recommendation models while preserving user privacy. By leveraging local data on users’ devices, FL enables the creation of personalized models without the need for centralizing sensitive user information. User behavior data, such as browsing history, app usage, and location information, can be utilized for training local models, which are then aggregated to generate global ad recommendation models [22,23].

#### 3.2.2. Contextual Advertising

Contextual advertising involves delivering ads based on the context of the user’s current activity or environment. FL enables the training of contextual ad targeting models using data distributed across devices or edge servers. Local models can be trained on context-specific data, such as search queries, website content, or real-time environmental data, while ensuring data privacy. Aggregating these models allows for the creation of context-aware ad-targeting models.

#### 3.2.3. Fraud Detection and Click-Through Rate Prediction

FL can be applied to detect fraudulent ad clicks and predict click-through rates (CTRs) accurately. By training local models on user engagement data, such as ad clicks, impressions, and historical conversion rates, FL enables the creation of fraud detection and CTR prediction models without exposing individual user data. The aggregated models effectively capture patterns and anomalies, enhancing fraud detection and ad targeting accuracy.

#### 3.2.4. Real-Time Ad Campaign Optimization

FL can facilitate the real-time optimization of ad campaigns by leveraging distributed data sources. Local models trained on real-time user interactions, such as ad views, clicks, and conversions, can be aggregated to generate global optimization models. This enables advertisers to dynamically adjust ad targeting, creatives, and bidding strategies in response to changing user behavior and campaign performance, without compromising data privacy [24].

## 4. The AROUND System Description

AROUND is a social network that prioritizes small- and medium-sized businesses through an intelligent advertising platform. The primary aim of AROUND is to facilitate meaningful connections between customers and traders by tailoring advertisements to create personalized shopping experiences. This approach aims to boost potential business interactions for both parties involved. As of now, the AROUND system boasts a user base of over 3 million users, with one million users actively engaged. Additionally, more than 12,000 beacons have been deployed to support their advertising efforts.

**Figure 1:**
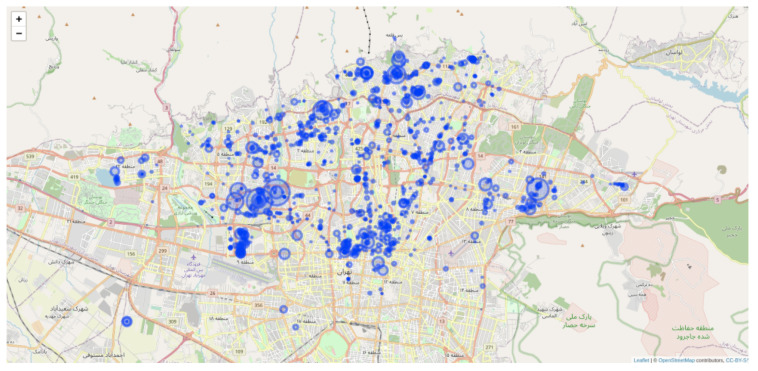
Map of current beacon deployment in the city of Tehran.

The AROUND system classifies its users into two distinct categories: traders and customers. Traders are essentially business owners who collaborate with the AROUND platform to provide personalized offers and advertising choices tailored to specific user profiles. To enable localized interactions with their clientele, traders deploy beacons in various locations. These beacons facilitate seamless communication and engagement with their customers within proximity. As depicted in Figure 1, the arrangement of beacons in Tehran city is illustrated, with over 800 registered traders and a deployment of more than 3000 beacons across the city.

**Figure 2:**
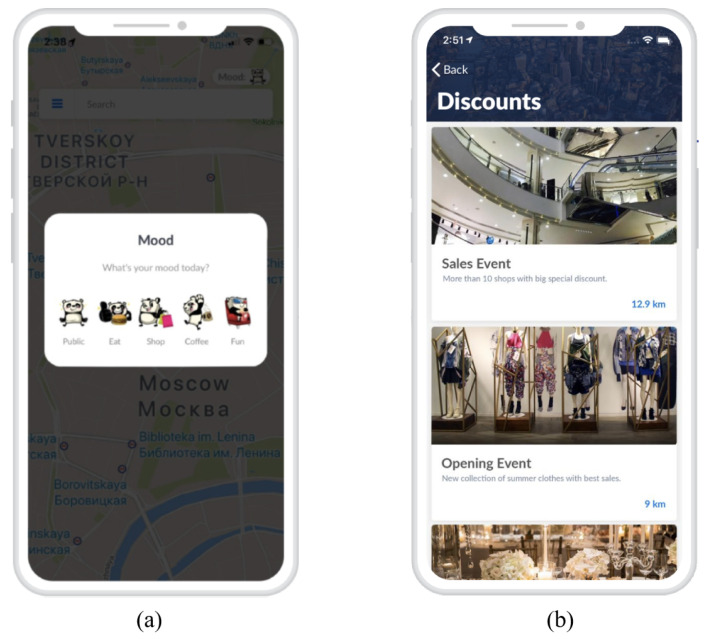
Screenshot of the mood selection (**a**) and sample advertisement (**b**) in AROUND.

Conversely, customers refer to individuals who utilize the app and desire to engage in social networking, share experiences with other users, and derive advantages from a customized shopping experience. During the registration process, customers establish their profile and have the option to select from five distinct moods on a daily basis as depicted in Figure 2a. The main service provided to AROUND users is the recommendation of the best advertising based on their historical behavior, which is determined by analyzing their in-app behavior and current location, and combined with their current defined mood. As an example, Figure 2b shows an advertisement sample.

**Figure 3:**
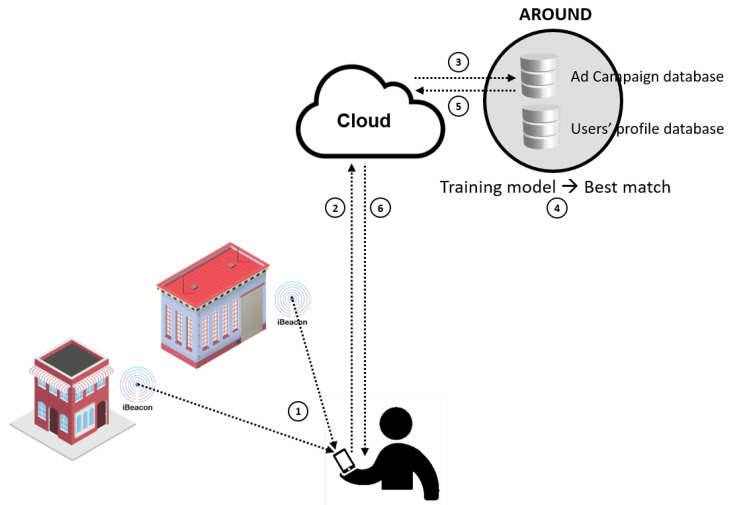
Basic operation of the AROUND system.

The operational process of the existing AROUND system is delineated in Figure 3. Numerous beacons are strategically positioned within the registered establishments, including shops, museums, or other commercial entities. Upon a registered customer nearing one of these business, the smartphone application identifies the beacon (1) and establishes a connection with the AROUND server (2) located in the cloud, relaying the current location of the customer (determined from the device’s location and the beacon’s range status). Leveraging these data in conjunction with the client profile, the AROUND system applies the trained model to determine the optimal advertisement for the customer (3–4–5). Subsequently, personalized promotions and discounts are conveyed to the customer (6) and displayed on the smartphone application.

The process of making a decision is as follows. A request is dispatched to a docker instance within a Kubernetes orchestration system for every user ranged by a beacon [25]. A clustering model is employed to categorize the request into nine primary classifications, supplemented by predefined scenarios for each category. The system takes into account various factors such as users’ behaviors, age, historical activities, and interactions with other users. Based on the user’s current disposition, the trained model recommends the most suitable category and selects a personalized advertisement. The AROUND system’s databases and operational framework are presently centralized in the cloud.

## 5. Data Model Architecture Overview

Building upon the insights gained from the previous section, we now introduce our proposed architecture aimed at addressing existing limitations and shortcomings. Implementing a data architecture for smart advertising using FL presents unique challenges that need to be addressed to ensure efficient and effective operations. The following challenges are crucial considerations in designing the data architecture:

- Data Authenticity and Validity: Ensuring the authenticity and validity of input data from clients is essential to maintain the integrity of the advertising models. Data validation mechanisms need to be implemented to verify the accuracy and reliability of the data before they enter the learning algorithm. Techniques such as outlier detection, data quality checks, and data cleansing methods can help identify and mitigate issues related to data authenticity [26]. As a component of our proposed architecture, validation is considered on both data (data nodes in Section 5) and models (validator nodes in Section 5).
- Privacy and Anonymity: Protecting user privacy is of the utmost importance in smart advertising. Data architecture should incorporate privacy-preserving mechanisms to ensure that user data are transmitted anonymously and independently of their identities. Techniques like differential privacy, secure aggregation, and encryption can be employed to safeguard user information while still enabling effective model training.
- Early Removal of Unwanted and Fake Data: Smart advertising data often contain noise, outliers, and fake data that can degrade the performance of the learning algorithm. To mitigate this issue, the data architecture should incorporate mechanisms for the early removal of unwanted and fake data on the client side. This can involve local data validation checks, anomaly detection techniques, and collaborative filtering methods to filter out unreliable data before they are used for model training.
- Computational Overhead: Minimizing computational overhead on the client side is crucial to ensure efficient and lightweight participation of devices in the FL process. Resource-constrained devices, such as mobile phones or IoT devices, may have limited processing power and energy constraints. Optimizations like model compression, pruning, and quantization can be applied to reduce the computational burden on client devices while maintaining model accuracy.
- Network Resource Consumption: In FL-based smart advertising, network resource consumption on the client side needs to be minimized to reduce communication costs and latency. Strategies like intelligent sampling, data prioritization, and adaptive communication protocols can be employed to optimize network resource utilization. These approaches aim to reduce the amount of data transferred between client devices and the central server, improving overall efficiency and responsiveness. In our proposed architecture, the distribution of roles among nodes obviates the necessity to disseminate the global model to all nodes, leading to a reduction in network resource consumption through various optimizations.

**Figure 4:**
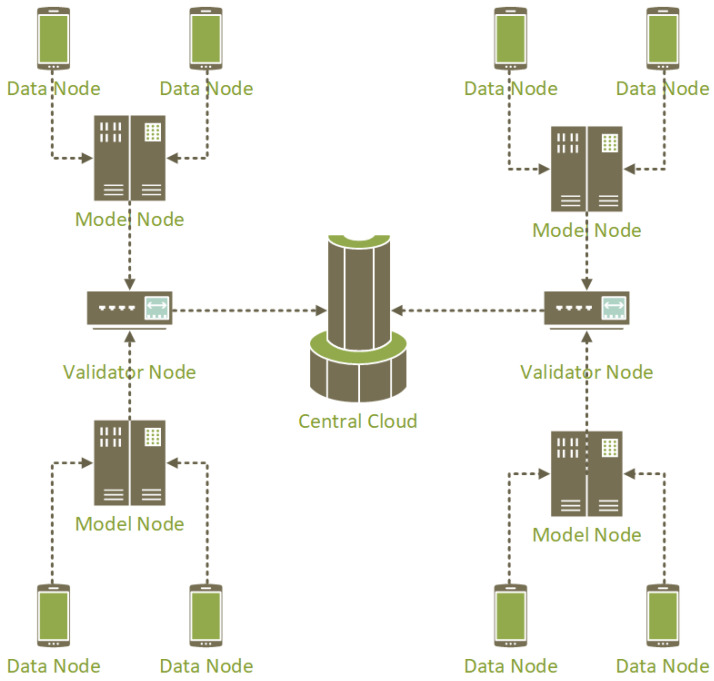
Overall architecture.

To tackle the challenge of minimizing network resource consumption in FL-based smart advertising, an effective strategy involves optimizing resource usage on the client side. The main idea to collect and integrate data according to all considered criteria is to use a semi-random assignment method. We describe an outline of this idea in this section. Figure 4 shows a rough overview of the main idea of this research.

In this architecture, after pinging the central cloud and obtaining the connection quality, each node sends a request to assign a role to the corresponding node to the central cloud. Model node, data node, and validator node are possible values for these roles.

The central cloud assigns this role due to the following features as well as a semi-random mechanism:

- Mobile device computing power;
- Quality of connection to the central cloud (ping, latency, etc.);
- History of relevant nodes in different roles.

In fact, only a fraction of nodes (for example, 30%) are selected as model nodes, which are responsible for building internal models. And a smaller fraction (for example, 10%) of the nodes are selected as validator nodes, which are responsible for validating the models and data obtained from other nodes.

Based on this, the main optimization concerns in the data model can be satisfied. Because all nodes are not always used for modeling and calculations, the average consumption of resources can be reduced in this way. Also, data loss is reduced because the central cloud also examines the quality of the connection in the selection of model nodes.

To assign a role (data node, model node, or validator node) to a node based on the given inputs and maintain the desired ratio, follow this process:

1. Collect the inputs for a node:
  - History of roles as a list: This list contains the previously assigned roles of the node.
  - Ping to the central server: The round-trip time (RTT) between the node and the central server.
  - Latency to the central server: The delay in communication between the node and the central server.
  - CPU Series: The specific series or model of the CPU on the node.
  - CPU Frequency: The clock speed of the CPU on the node.
  - CPU Ideal amount: The desired or optimal CPU utilization for the node.
2. Determine the role assignment:
  - Calculate the number of data nodes (N), model nodes (N/C), and validator nodes (N/(C*C)) based on the desired ratio. Here, C represents a constant factor.
  - Check the history of roles for the node. If the node has been previously assigned as a Model Node or Validator Node, exclude those roles from the possible assignment options.
  - Evaluate the inputs to determine the most suitable role for the node. Consider factors such as the following:
    – CPU Series and Frequency: Nodes with higher CPU capabilities may be better suited as model nodes.
    – Ping and Latency to the central server: Nodes with low ping and latency values may be preferred as validator nodes for faster validation.
    – CPU Ideal amount: Nodes with an ideal CPU amount can be assigned as data nodes.
3. Assign the role:
  - Based on the evaluation, assign the node the most suitable role considering the inputs and the desired ratio.
  - Update the history of roles for the node with the newly assigned role.

By following this process, you can assign roles to the nodes while maintaining the desired ratio of data nodes, model nodes, and validator nodes. Adjusting the parameters and evaluation criteria in step 2 can help further refine the role assignments based on specific requirements and considerations.

The methodology in the research paper integrates system performance optimization with user behavior monitoring to consider holistically the effectiveness of smart advertising strategies. The system performance optimization is achieved through the application of FL, which ensures efficient data flow, security, and privacy while maintaining high performance. User behavior monitoring is crucial for understanding audience preferences and delivering personalized ads that resonate with the target audience, thereby increasing engagement and conversion rates.

The combination of these two aspects is driven by the need to provide relevant and timely content to users, enhancing the overall ad experience and maximizing the impact of advertising efforts. By leveraging data analytics and artificial intelligence algorithms, advertisers can extract valuable insights from user data, anticipate user preferences, and deliver tailored ads that are more likely to generate interest and action. The methodology’s focus on system performance and user behavior monitoring aims to create a symbiotic relationship between efficient ad delivery and user engagement, ultimately optimizing the effectiveness of smart advertising campaigns.

## 6. The Core Recommender System

The core recommender system is built using a FL approach, where models are trained at both the edge side and the central cloud level. The goal is to create an effective recommendation system by considering the behavior of users in different contexts and incorporating random effects. The primary emphasis in this system lies in the accuracy of user selection, alongside other crucial parameters such as click-through rate (CTR) and user engagement.

At the edge side, each location or zone has its own model that takes into account the specific behavior patterns of the users in that zone. These models capture the details of different user behaviors through the coefficients of significant variables. The coefficients represent the impact of specific factors on user preferences or actions.

Simultaneously, there is a central level model trained using the gathered data. This model estimates the additive part of the multinomial model, which represents the random effects in multilevel models. The random effects capture the intercorrelation within subjects at each level, indicating how users in the same combination of zone and iBeacon are influenced by each other’s behavior.

When migrating data from the edge to the cloud, there are considerations for privacy and data protection. One approach is to anonymize each data point before sending it to the main cloud. Alternatively, the main cloud can wipe the data used for each successfully converged model, ensuring that sensitive information is not retained.

The accuracy and performance of the models on different edge sides are crucial when estimating the random effect part of the model. A predefined threshold can be set to ensure that only performant models are considered in the estimation process.

The deployment levels mentioned earlier are not related to the deployment of the system but rather to the intercorrelation between users in the same combination of zone and iBeacon. The significant multilevel coefficients indicate the presence of intercorrelation within subjects at each level, reflecting how users in the same socio-economic iBeacon zone exhibit similar behaviors.

To start, a robust model is deployed in the main cloud to serve users and classify their behavior. The advertisement campaigns are designed to be effective at their maximum level based on the labels derived from a previous clustering method with a custom distance function.

A baseline multinomial logit model is used, with the cluster representing fresh installers as the baseline class. The effect of each edge side is considered random since the sampled iBeacon devices represent a subset of all future iBeacon devices that will be ranged. Additionally, a last location categorical variable is introduced, dividing Tehran into 22 districts and further grouping them into five separate socioeconomic zones. Within each zone, three edge sides are selected.

The model training in the central cloud is performed using a semi-unbalanced labeled dataset of one million instances. For estimating the random effect and its standard error, the PQL (Penalized Quasi-Likelihood) method with Laplace approximation is employed. On the other hand, the estimation of fixed effects and other covariates is performed using Maximum Likelihood estimation. The training process involves an iterative algorithm that may be time-consuming, but for faster training, Restricted Maximum Likelihood can be considered an alternative.

**Figure 5:**
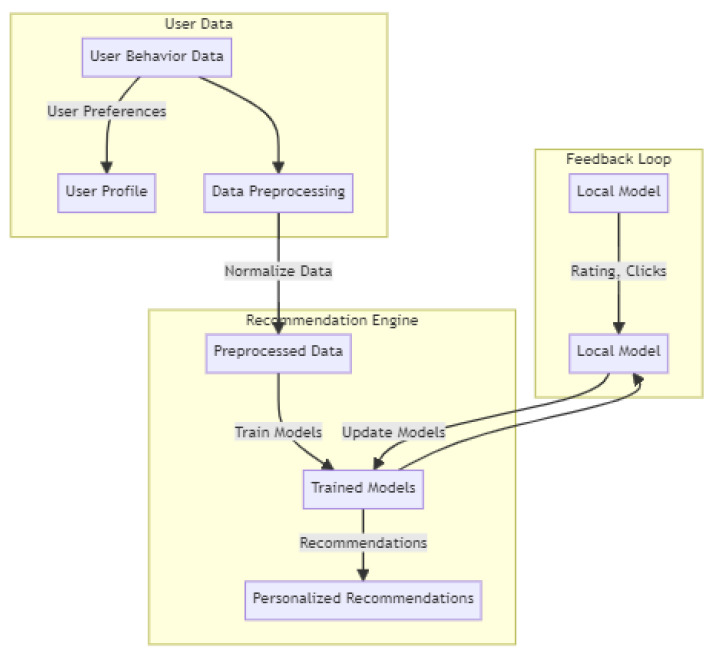
Overview of the core recommender system.

Figure 5 provides a more detailed illustration of the components in the core recommender system. The recommendation engine is placed on the central cloud, and two other components are in the edge side. The user data component is especially equal to devices (iBeacons) and the feedback loop is implemented in the level of zones which are discussed above.

The labeling for this phase of modeling is adjusted to make user activity more visible. Cluster 1 represents fresh installers, while cluster 9 represents the most active and loyal users. The estimation methods used in this model are iterative algorithms that typically converge after 6 to 7 iterations.

Overall, this subsection outlines the core recommender system, explaining the FL approach with models at both the edge side and central cloud level. It also highlights the considerations for data migration, model performance, and the training process in the central cloud.

**Figure 6:**
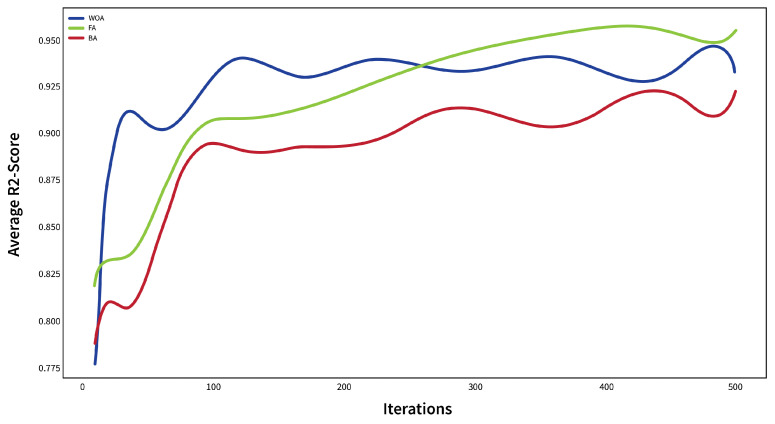
The impact of the number of iterations on the aggregation process.

For the implementation of the core recommender system, the first crucial step involves selecting the most suitable option among the candidate meta-heuristics for performing aggregation. To achieve this, an experiment is designed, varying the number of iterations of meta-heuristics used in the aggregation process. Figure 6 illustrates the results of this experiment, showcasing the performance of different aggregation approaches. On the other hand, Table 1 shows the minimum score in the number of different iterations.

Several candidate meta-heuristics for aggregation in FL systems were used in various applications. Here, and through experimentation, it became evident that the Whale Optimization Algorithm (WOA) [27] exhibited notably faster convergence rates, thereby facilitating quicker and more effective optimization processes. Additionally, it consistently showcased superior performance compared to other algorithms, such as the Firefly Algorithm (FA) [28] and BAT Algorithm (BA) [29], especially when the number of iterations for the FA was increased. However, despite the improved performance of the FA with increased iterations, its convergence rate was relatively slower in comparison to the WOA.

By choosing the WOA, the recommender system achieves remarkable stability, which is particularly valuable in real-world scenarios. The faster convergence of the WOA allows the system to achieve high accuracy with a minimum number of iterations, offering reliable and consistent performance. This stability ensures that the recommender system can handle diverse datasets and varying user preferences, making it more robust and practical for deployment.

On the other hand, Table 1, which shows the minimum score in the number of different iterations, confirms the stability of the WOA.

In brief, the core recommender system in this study leverages logistic regression for its local models. These models are aggregated using the WOA with a population size of 50 individuals across 10 iterations. Additionally, 120 iterations are conducted for aggregation within the FL process. These settings are selected based on the previous facts and figures in this section.

## 7. Results and Discussion

In this section, we delve into a comprehensive evaluation of the proposed data architecture, focusing on its performance, efficiency, and suitability for smart advertising contexts. Our experimental approach is designed to align closely with the architecture’s key features and objectives, providing insights into its effectiveness in real-world scenarios.

To ensure clarity and consistency, our experiments are organized to address specific aspects of the architecture that we aim to demonstrate. We have carefully structured our experimental setup to evaluate critical parameters such as network traffic, resource consumption (related to FL), and the accuracy of the recommender system. By aligning our experiments with these core elements, we aim to provide a comprehensive understanding of the architecture’s capabilities and limitations.

First, we start with results related to architecture and FL. The primary aim of quantifying the resource consumption time is to ascertain the ratio of time allocated by the devices engaged in processing tasks within the given architecture per hour. This metric can serve as a criterion for evaluating the efficacy of the proposed architecture in distributing computational tasks across devices. Second, the amount of communication overhead is measured and compared in the case of the proposed architecture and the use of the central server. These tests are performed in the real environment (AROUND).

Subsequently, it is imperative to assess the efficacy of the suggested approach in relation to the advertising recommender system. The performance of the models constructed within the recommender system is conducted in a real environment, with a focus on the manner in which the outcomes of the internal models are aggregated (aggregation method). Then, the click rate is compared with the proposed method and the central server mode as well as normal (random) advertisements. Similarly, the amount of attention users pay to selected advertisements is measured under the title of user engagement. Lastly, the rate at which users dismiss ads or leave the system is evaluated (Opt-Out users).

In addition to the mentioned cases, the results of the proposed architecture in a simulated environment are also evaluated and compared with the real environment, which is reviewed in the last section of the results (under the seventh section of the results). In this way, considering that we can increase the number of devices in the simulated environment without restrictions, we obtain a better insight.

### 7.1. Resource Consumption Time

In order to evaluate the whole project in terms of resource consumption, we designed an experiment with three different levels of device populations: 25, 50, and 100. The reason for choosing these numbers, which are small numbers, is that we need to obtain permission from the user on our device to track the amount of resource consumption. Therefore, this experience is checked only with a limited number of devices. This experiment directly illustrates the effect of the proposed architecture and shows how this architecture reduces the mean time each device is used in this framework.

It should be noted that this experimental result is different from the previous result regarding CPU consumption in two ways. First, in this experiment, the entire system is evaluated, and secondly, the time used by certain devices is checked as an average.

The results in Table 2 show that increasing the device population from 25 to 100 significantly reduces the mean device usage time per hour, from 24,732 ms to 8730 ms, a 64% decrease. This demonstrates the efficiency of the proposed architecture in distributing workloads, making it scalable and reducing the per-device resource consumption. Consequently, as more devices participate, the system becomes more efficient, lowering energy consumption and improving user experience.

### 7.2. Communication Overhead

Edge-based environments have some communications between devices, and FL requires communication between the central server and user devices. As one of the evaluations of our architecture, we have to analyze the communication overhead and its impact on the overall system performance.

**Figure 7:**
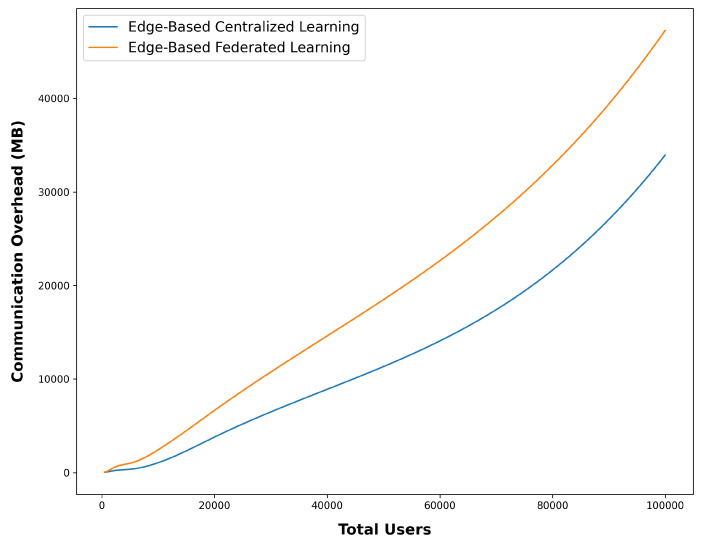
Comparison of communication overhead in Central and FL.

For this purpose, through a calculation function, the amount of data exchanged for different situations of users who use the system was checked in two situations. The edge-based mode with central learning and the edge-based mode with FL were investigated, and the results are visualized in Figure 7.

Upon analyzing the outcomes of this experiment, it is evident that the communication overhead in federated learning (FL) is inherently higher compared to centralized learning. However, the rate at which this overhead increases with the number of users is not significantly different from the central learning mode. For instance, when considering 50,000 users, the communication overhead is approximately 10,000 megabytes when utilizing edge-based federated learning, whereas it exceeds 15,000 in the baseline central cloud. While it is possible to conduct separate research to further optimize this communication overhead, the current levels of overhead appear to be acceptable for the purposes of this study.

The manageable increase in communication overhead is a notable finding, as it showcases the efficiency of FL in distributing the learning process across decentralized devices. This distributed approach allows devices to collaboratively learn while minimizing the need for extensive data transfers to a central server. The marginal increase in the communication overhead suggests that FL can scale effectively even with a larger number of users, making it a viable option for real-world applications involving a diverse user base.

While the current study does not delve deeply into communication optimization techniques, future research endeavors can certainly explore strategies to further enhance communication efficiency. These strategies may encompass techniques like model compression, quantization, and differential privacy, which aim to reduce the amount of information exchanged while maintaining model accuracy and data privacy. By integrating these techniques, it is possible to mitigate the potential challenges posed by increased communication overhead as the FL system scales up.

Moreover, the acceptable levels of communication overhead observed in this study are encouraging for industries that are increasingly adopting FL. Sectors like healthcare, finance, and manufacturing, where data privacy and regulatory compliance are paramount, can benefit from the ability of FL to maintain data on local devices while achieving meaningful model updates. This not only minimizes privacy risks but also fosters collaboration and knowledge-sharing in a secure manner.

In summary, while communication overhead does experience a natural increase in FL, the study demonstrates that this increase is manageable and comparable to centralized learning scenarios, even as the number of users scales up. The potential for further optimization and the promising implications for industries with stringent privacy requirements make FL a compelling approach for a wide range of applications.

### 7.3. Model Performances

An essential aspect of the experiment in this research is the evaluation of the models’ performance and accuracy, which provides valuable insights into the proposed method. The experimental design aims to showcase the accuracy of the models, and threestatistical evaluation criteria are utilized for this purpose:

- F1-Score;
- Nagelkerke.

The results from Table 3 and Table 4 indicate that the proposed architecture significantly improves model performance, especially when using meta-heuristic aggregation. For instance, predicting Class 1 with the proposed method achieves an almost perfect F1-Score of 0.99785, compared to 0.87785 with default aggregation. Additionally, the Nagelkerke values show improvements, with Class 1 reaching 0.99950 using meta-heuristic aggregation, demonstrating near-ideal fit. These enhancements highlight the utility of the proposed architecture in achieving high prediction accuracy and better model performance, making it a robust solution for classification tasks. This efficiency in performance underscores the framework’s ability to provide precise and reliable predictions, which is crucial for practical applications.

### 7.4. Click Rate

One of the most important parameters in any advertising system, be it an ‌ordinary online ad or smart ad, is the click rate. To measure this success rate in smart advertising, we used the conventional CTR parameter.

The CTR (click-through rate) is a metric used in online advertising to measure the percentage of users who click on a specific advertisement, relative to the total number of users who view the ad:

(1) $$CTR=\frac{\mathrm{Number}\;\mathrm{of}\;\mathrm{Clicks}\;\mathrm{on}\;\mathrm{the}\;\mathrm{Ad}}{\mathrm{Number}\;\mathrm{of}\;\mathrm{Ad}\;\mathrm{Impressions}}\times 100\%$$

In order to carry out this evaluation, the CTR parameter was measured for 50 ads in three different modes, and the average mode is shown in Table 5. These three options are the normal mode of advertisement selection—using the machine learning model centrally and using the entire architecture provided with the help of FL.

Also, in Table 6, these results are shown separately for two types of advertisements related to e-commerce and entertainment. This table gives us an idea about the success of the presented method in e-commerce compared to entertainment. This fact can be due to the fact that according to the considered parameters, customized ads better represent the interests of users in shopping.

E-commerce advertisements inherently require a higher degree of personalization to effectively engage users. Users visiting e-commerce platforms are often looking for specific products or deals, and personalized ads aligned with their preferences tend to be more relevant and influential. The parameters considered in the study, such as user browsing history, search queries, and purchase behavior, play a critical role in tailoring ads that cater to individual shopping needs.

In contrast, entertainment-related advertisements might be more challenging to personalize to the same extent. Entertainment preferences can vary widely among users and can be influenced by mood, cultural factors, and diverse interests. As a result, achieving the same level of personalization for entertainment ads may require a more complex understanding of user behavior and preferences, incorporating factors beyond the ones considered in this study.

The discrepancy in the success of the presented method between e-commerce and entertainment domains underscores the importance of context and user intent. E-commerce advertisements are more likely to align with users’ immediate goals, making personalized recommendations highly effective. Meanwhile, entertainment ads might benefit from more advanced techniques that capture subtler aspects of user behavior and preferences, such as sentiment analysis, social interactions, and content consumption patterns.

To enhance the performance of the presented method for both e-commerce and entertainment domains, future research could explore combining a wider array of user data and employing more sophisticated machine learning algorithms. Additionally, the dynamic nature of user interests and the evolving landscape of advertising platforms call for adaptive strategies that can continuously learn and adapt to changing user behaviors and trends.

The results in Table 5 show that the proposed architecture and federated ML approach significantly improve CTR, achieving an average of 53.9%, compared to 48.1% with central ML and 43.2% with ordinary advertisements. Table 6 further demonstrates this improvement across domains: e-commerce ads saw a CTR increase to 57.9% from 50.3% (central ML) and 46.4% (ordinary ads), and entertainment ads increased to 50.7% from 46.2% (central ML) and 40.5% (ordinary ads). These enhancements highlight the method’s effectiveness in boosting user engagement and ad relevance, especially in e-commerce, where personalized ads align closely with user shopping interests, ultimately enhancing advertising campaign efficacy.

### 7.5. User Engagement

FL is expected to enable better personalized ad targeting, leading to increased user engagement with the advertisements. This could be measured through metrics such as the time spent on the ad and the number of interactions with the ad.

**Figure 8:**
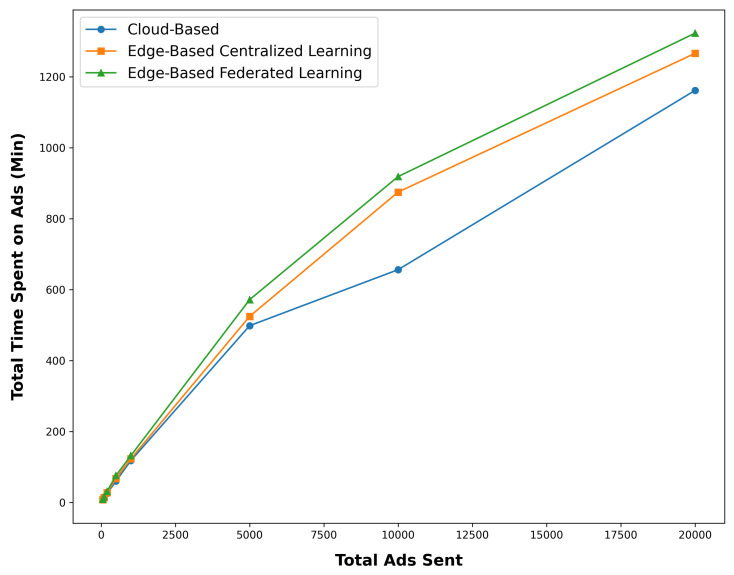
Time spent on the ad in different settings.

In this experiment, the ads sent are 25–30 s long videos, and the maximum time spent by the user is 30 s for each ad. The results of this experiment are shown in Figure 8. By examining this figure, we can see that the complete architecture presented increases the amount of attention users pay to advertisements.

**Figure 9:**
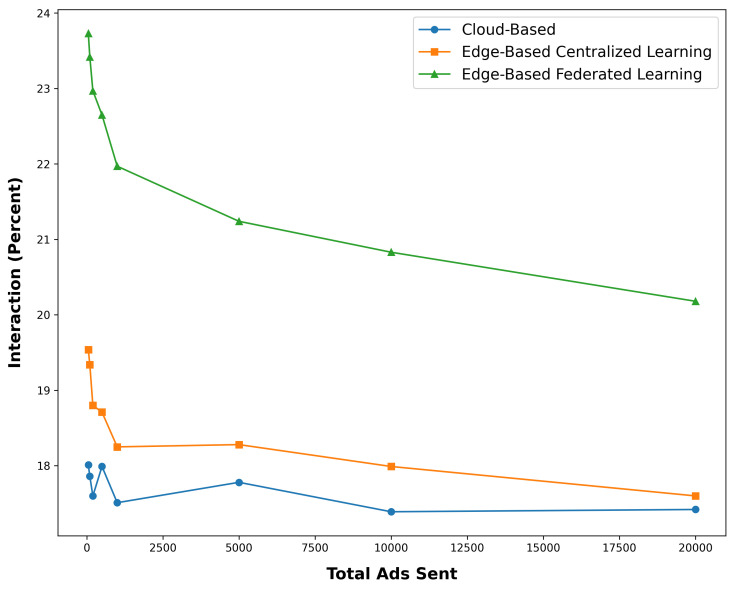
The percentage of interaction on the ad in different settings.

In general, in Figure 9, this superiority can be seen in the FL mode in the edge-based mode. The justification of this superiority is that in the case of the central cloud, the user’s profile information must first be collected in the central cloud and then processed, and advertisements selected with its help. This issue slows down the process a bit, and as a result, the user’s needs and general profile are not up to date for selection. In edge-based mode with central learning, although this update is performed faster and this improves the result a little, the modeling process is still central and requires time. But in the edge-based mode, with FL, the user’s features are updated quickly and the advertisement selection model is built in real time. Therefore, the general attention of users is significantly improved according to their features.

Figure 8 and Figure 9 show that the proposed architecture significantly increases user engagement. In FL mode, the time spent on ads shows almost 5% increase, and interaction rates exceed 23%, much higher than in other settings. These results highlight the utility of FL in enhancing personalized ad targeting by updating user profiles in real time, leading to more relevant ad selection and higher engagement. This increased engagement is beneficial for advertisers, suggesting higher potential conversion rates. Future research could explore the scalability of this architecture for diverse ad formats and investigate the relationship between user engagement and business outcomes like conversion rates and brand recall, offering a deeper understanding of the impact of FL on smart advertising.

### 7.6. Opt-Out Users

Another significant metric that can be employed to gauge the effectiveness of advertisements and user targeting is the rate at which users opt out of viewing ads or exit the entire system. This comprehensive metric provides insights into the overall user satisfaction and engagement with the ads presented.

**Figure 10:**
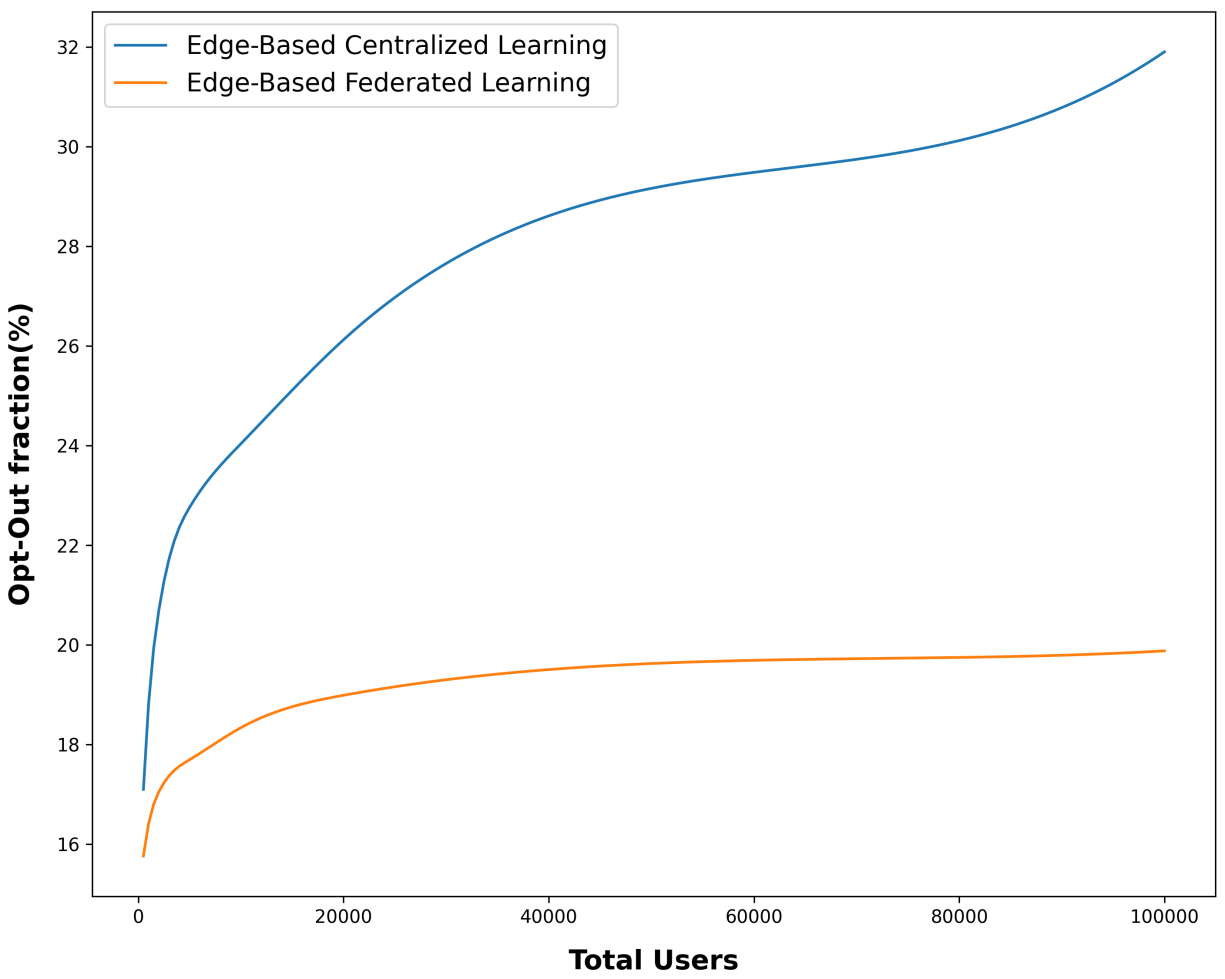
Comparison of opt-out fraction in Central and FL.

The results obtained for this criterion, as depicted in Figure 10, underscore a notable trend. Specifically, in the presented FL mode, the rate at which users opt out or exit the system exhibits a growth rate that is comparatively lower than what is observed in the central learning mode. This observation carries significant implications and contributes to confirming the relative success of the proposed method in the context of user engagement and retention.

The decreased growth rate of user opt-out in the FL mode can be attributed to the benefits inherent in personalized advertising. By tailoring ads to individual preferences, needs, and behaviors, users are more likely to find the advertisements relevant and valuable. This relevance enhances user experience and reduces the likelihood of users opting out due to irrelevant or intrusive ads.

Moreover, the distributed nature of FL, where model updates are performed locally on devices without transmitting raw data, contributes to enhancing user trust and privacy. The emphasis on data security and local processing ensures that user data remain on their devices, alleviating concerns that users might have about their information being used for targeting without their consent.

The favorable trend in the opt-out rate aligns with the broader goals of advertisers—to create engaging ad experiences that resonate with users and encourage their continued participation. The ability of FL to maintain user engagement while respecting their privacy underscores its potential for establishing a more harmonious and ethical advertising ecosystem.

In the edge-based federated learning (FL) setting, the percentage of users who choose to opt out remains below 20%, even as the number of users increases by 100,000. In contrast, in the baseline setting, there is a significant increase in this percentage.The relatively slower decline in the rate at which users opt out, as observed in the FL mode, is a compelling indication of the effectiveness of the method being presented. It demonstrates the efficacy of personalized advertisements as well as the beneficial effects of privacy-preserving and user-centric methods facilitated by FL.

### 7.7. Simulated Results

Table 7 and Table 8 summarize the classification of users based on their activity in smart advertising for the the simulated and real Around environments respectively. In both tables, N is the number of the nodes and C represents a constant factor, as described in Section 5.

**Figure 11:**
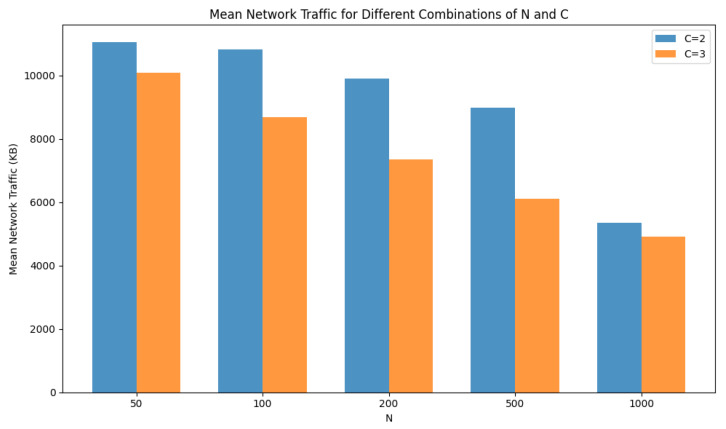
Mean network traffic for different combinations of N and C.

The mean network traffic, measured in kilobytes (KB), as shown in Figure 11, provides insights into the amount of data transmitted over the network in the simulation. From the results, we observe that as the number of nodes (N) increases, the mean network traffic tends to decrease. This suggests that with a larger number of nodes, the network load per node decreases.

The F1-Score serves as a pivotal metric for assessing the precision and recall of a classification model, particularly in instances characterized by class imbalances. Analogous to the interpretation of a high F1-Score value in regression models denoting superior fit, achieving F1-Scores proximate to 1 signifies exemplary accuracy in discerning between classes. This underscores the model’s adeptness in making precise predictions regarding user activity based on the input features, thereby affirming its efficacy in classification tasks.

**Figure 12:**
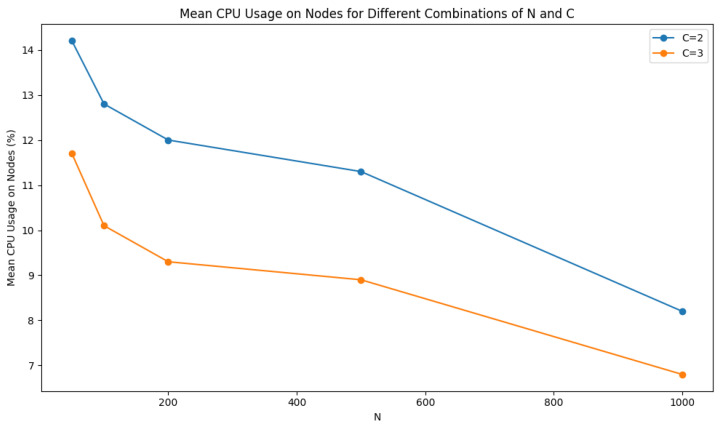
Mean CPU usage on nodes for different combinations of N and C.

The mean CPU usage on nodes, expressed as a percentage, Figure 12, provides insights into the computational load experienced by the nodes in the simulation. From the results, we observe that as the number of nodes (N) increases, the mean CPU usage on nodes tends to decrease. This indicates a lower computational load per node as the network scales.

Overall, these results demonstrate the relationship between the number of nodes, network traffic, F1 values, and mean CPU usage on nodes, providing valuable insights into the performance of the classification system for smart advertising.

### 7.8. Summary of Findings

Notably, the system was able to achieve a remarkable reduction of over 50% in both network traffic and average CPU usage, even with a twenty-fold increase in user count. In addition to evaluating network traffic and resource consumption, the research delved into a comprehensive assessment of the proposed data architecture’s performance, efficiency, and suitability for smart advertising contexts. The experimental setup was carefully structured to address specific aspects of the architecture and provide insights into its effectiveness in real-world scenarios.

The research findings highlight the scalability and high performance of the proposed architecture in smart advertising environments. Metrics such as click rate, user engagement, and other parameters were evaluated to demonstrate that the advantages of the architecture do not compromise the accuracy of smart advertising.

These findings underscore the architecture’s capacity to scale efficiently and maintain high performance, making it a valuable contribution to the digital marketing landscape and the field of federated learning.

## 8. Conclusions

This research presents a detailed architecture for migrating a smart advertising system from the central cloud to the edge-based and FL mode. The architecture is comprehensive and can be examined from multiple perspectives. The research paper introduces an innovative data architecture specifically tailored for smart advertising applications, emphasizing the enhancement of data flow, performance, security, and data privacy. The primary approach utilized in this architecture is federated learning (FL), which guarantees the integrity and confidentiality of data by promptly discarding irrelevant or fraudulent information. This approach is essential in the context of smart advertising, where maintaining the accuracy and confidentiality of data is of the utmost importance.

The data model proposed in the architecture utilizes a semi-random role assignment strategy based on various criteria to efficiently collect and amalgamate data from different nodes. These nodes include model nodes, data nodes, and validator nodes, with each node’s role determined by factors such as computational capability, interconnection quality, and historical performance records. This strategic role assignment helps in optimizing resource utilization and minimizing data loss, thereby enhancing the overall efficiency of the architecture.

A key feature of the proposed architecture is the selective engagement of a subset of nodes for modeling and validation purposes. This selective engagement further contributes to resource optimization and ensures that only relevant nodes are involved in the data-processing tasks, thereby improving the overall performance of the system.

The effectiveness of the proposed architecture was evaluated using the AROUND social network platform as a real-world case study. The results of both simulated and real implementations of the architecture demonstrated its potential to significantly reduce network traffic and average CPU usage while maintaining the accuracy of the FL model.

The findings reveal several important insights. Firstly, the mean network traffic decreases as the number of nodes increases, indicating that the proposed system efficiently handles data transmission with larger node populations. The high F1-Score values indicate a strong fit of the classification model, affirming its ability to accurately capture the relationship between user activity and input features.

Furthermore, the mean CPU usage on nodes decreases with an increase in the number of nodes, implying a lower computational load per node in larger networks. This scalability is vital for accommodating more devices and users while maintaining system performance.

Comparing model performances, the proposed federated aggregation method outperforms the default aggregation, resulting in improved prediction accuracy. The resource consumption time experiments demonstrate the system’s efficiency and its ability to handle larger device populations with reduced resource utilization.

In terms of user engagement, the proposed architecture with edge-based FL stands out, leading to higher click-through rates and increased user attention to advertisements. The real-time updates and personalized ad selection contribute to enhanced user experiences.

Also, the results showcase that the FL approach leads to a lower growth rate of user opt-out compared to the central learning mode, indicating improved user engagement and satisfaction with personalized ads. This outcome underscores the method’s potential to create more relevant and privacy-respecting advertising experiences, enhancing the overall effectiveness of ad campaigns while fostering user trust.

In summary, the proposed method offers a promising approach for smart advertising classification, with the potential to enhance system performance, scalability, and user engagement. The combination of edge-based FL, real-time updates, and personalized ad targeting presents a powerful solution for advertisers to reach their target audience effectively.

It is essential to acknowledge that this study’s findings are based on simulated results, and further real-world experiments and deployments would be necessary to validate the effectiveness and practicality of the proposed method in actual smart advertising systems.

Overall, the research demonstrates the significant strides made in the field of smart advertising, leveraging advanced techniques like FL and edge computing to create more efficient, personalized, and engaging advertising experiences for users while ensuring privacy and data security.

Building upon the insights gained from this research, several avenues for future work emerge. Firstly, exploring the impact of different FL optimization techniques could provide a deeper understanding of their effectiveness in improving model accuracy and convergence rates. Investigating hybrid models that combine edge-based FL with centralized cloud processing could strike a balance between local efficiency and global optimization.

Further research could delve into addressing the challenges of handling heterogeneous devices in a FL setup. Developing adaptive strategies that account for varying computational capabilities and connectivity of edge devices could optimize training across the network. Additionally, incorporating privacy-preserving mechanisms like secure aggregation and homomorphic encryption could strengthen data protection while sharing gradients for model updates.

Moreover, the exploration of multi-modal data integration, including social media interactions, sentiment analysis, and location-based insights, could lead to more comprehensive user profiles and enhanced ad personalization. Integrating user feedback loops to dynamically adjust model parameters and ad content based on real-time responses could further elevate user engagement.

## Figures and Tables

**Table 1:** Minimum F1-Score in different numbers of iterations.

Number of Iterations	Minimum Score				
	100	200	300	400	500
WOA	0.729	0.754	0.869	0.931	0.937
FA	0.751	0.729	0.852	0.896	0.923
BA	0.691	0.698	0.820	0.892	0.894

**Table 2:** Mean time of device usage per hour using the whole architecture.

Device Population	Mean Time of Device Usage Per Hour
25	24,732 ms
50	16,978 ms
100	8730 ms

**Table 3:** Impact of proposed architecture on model performance (default aggregation).

Metric	Class 1	Class 3	Class 5	Class 7	Class 9
Nagelkerke	0.89950	0.89853	0.87529	0.83734	0.85929
F1-Score	0.87785	0.87441	0.86411	0.86947	0.84383

**Table 4:** Impact of proposed architecture on model performance (meta-heuristic aggregation).

Metric	Class 1	Class 3	Class 5	Class 7	Class 9
Nagelkerke	0.99950	0.89853	0.87221	0.93684	0.98225
F1-Score	0.99785	0.92641	0.91451	0.95947	0.98322

**Table 5:** CTR in different conditions.

Test Case	CTR
Ordinary Advertisement	43.2%
Using Central ML	48.1%
Proposed Architecture and Federated ML	53.9%

**Table 6:** CTR in different business domains.

Test Case	E-Commerce	Entertainment
Ordinary Advertisement	46.4%	40.5%
Using Central ML	50.3%	46.2%
Proposed Architecture and Federated ML	57.9%	50.7%

**Table 7:** Results of the proposed method using different N and C values (simulated environment).

N	C	Mean Network Traffic (KB)	F1-Score	Mean CPU Usage on Nodes (%)
50	2	11,050	0.9823	14.2
100		10,825	0.9804	12.8
200		9901	0.9813	12.0
500		8997	0.9838	11.3
1000		5342	0.9829	8.2
50	3	10,098	0.9798	11.7
100		8680	0.9774	10.1
200		7344	0.9766	9.3
500		6109	0.9762	8.9
1000		4914	0.9769	6.8

**Table 8:** Results of the proposed method using different N and C values (around environment).

N	C	Mean Network Traffic (KB)	F1-Score	Mean CPU Usage on Nodes (%)
48	2	11,050	0.9723	14.4
87		9825	0.9794	12.8
160		8616	0.9769	8.8
48	3	10,098	0.9639	11.9
87		7631	0.9717	9.9
160		7370	0.9710	9.1

## Data Availability

Data presented in this study are available on request from the corresponding author.
